# Association of multiple metabolic and cardiovascular markers with the risk of cognitive decline and mortality in adults with Alzheimer’s disease and AD-related dementia or cognitive decline: a prospective cohort study

**DOI:** 10.3389/fnagi.2024.1361772

**Published:** 2024-04-02

**Authors:** Longjian Liu, Edward J. Gracely, Xiaopeng Zhao, Gediminas P. Gliebus, Nathalie S. May, Stella L. Volpe, Jingyi Shi, Rose Ann DiMaria-Ghalili, Howard J. Eisen

**Affiliations:** ^1^Department of Epidemiology and Biostatistics, Dornsife School of Public Health, Drexel University, Philadelphia, PA, United States; ^2^Department of Family, Community & Preventive Medicine, College of Medicine, Drexel University, Philadelphia, PA, United States; ^3^Department of Mechanical, Aerospace, and Biomedical Engineering, University of Tennessee, Knoxville, Knoxville, TN, United States; ^4^Department of Neurology, College of Medicine, Drexel University Philadelphia, Philadelphia, PA, United States; ^5^Department of Medicine, College of Medicine, Drexel University, Philadelphia, PA, United States; ^6^Department of Human Nutrition, Foods, and Exercise, Virginia Polytechnic Institute and State University, Blacksburg, VA, United States; ^7^Department of Mathematics and Statistics, Mississippi State University, Starkville, MS, United States; ^8^Doctoral Nursing Department, Nutrition Science Department, College of Nursing and Health Professions, Drexel University, Philadelphia, PA, United States; ^9^Clinical Research for the Advanced Cardiac and Pulmonary Vascular Disease Program, Thomas Jefferson University Hospital, Philadelphia, PA, United States

**Keywords:** Multiple biomarkers, metabolic and vascular disorders, association analysis, risk of cognitive decline, Alzheimer’s disease

## Abstract

**Background and objectives:**

There is a scarcity of data stemming from large-scale epidemiological longitudinal studies focusing on potentially preventable and controllable risk factors for Alzheimer’s disease (AD) and AD-related dementia (ADRD). This study aimed to examine the effect of multiple metabolic factors and cardiovascular disorders on the risk of cognitive decline and AD/ADRD.

**Methods:**

We analyzed a cohort of 6,440 participants aged 45–84 years at baseline. Multiple metabolic and cardiovascular disorder factors included the five components of the metabolic syndrome [waist circumference, high blood pressure (HBP), elevated glucose and triglyceride (TG) concentrations, and reduced high-density lipoprotein cholesterol (HDL-C) concentrations], C-reactive protein (CRP), fibrinogen, interleukin-6 (IL-6), factor VIII, D-dimer, and homocysteine concentrations, carotid intimal-medial thickness (CIMT), and urine albumin-to-creatinine ratio (ACR). Cognitive decline was defined using the Cognitive Abilities Screening Instrument (CASI) score, and AD/ADRD cases were classified using clinical diagnoses.

**Results:**

Over an average follow-up period of 13 years, HBP and elevated glucose, CRP, homocysteine, IL-6, and ACR concentrations were significantly associated with the risk of mortality in the individuals with incident AD/ADRD or cognitive decline. Elevated D-dimer and homocysteine concentrations, as well as elevated ACR were significantly associated with incident AD/ADRD. Elevated homocysteine and ACR were significantly associated with cognitive decline. A dose–response association was observed, indicating that an increased number of exposures to multiple risk factors corresponded to a higher risk of mortality in individuals with cognitive decline or with AD/ADRD.

**Conclusion:**

Findings from our study reaffirm the significance of preventable and controllable factors, including HBP, hyperglycemia, elevated CRP, D-dimer, and homocysteine concentrations, as well as, ACR, as potential risk factors for cognitive decline and AD/ADRD.

## Introduction

Alzheimer’s disease (AD) and AD-related dementias (AD/ADRD) are intricate neurological disorders that have a profound impact on millions of Americans, presenting some of the most significant healthcare challenges of the twenty-first century. In the United States (US), it was estimated that approximately 6.2 million adults aged 65 years and above were living with AD/ADRD in 2021, with projections indicating a staggering increase to approximately 14 million by the year 2060 (Tahami Monfared et al., 2022). The pathogenesis of AD/ADRD involves a complex interplay of various factors. Individuals experiencing cognitive decline (a precursor to dementia) and AD/ADRD exhibit a range of physiological alterations, including dysglycemia, dyslipidemia, endothelial dysfunction, vascular disorders, and chronic inflammation (Tahami Monfared et al., 2022). Despite these physiological alterations, the majority of researchers have primarily concentrated on genetics and protein concentrations, with limited attention given to applied epidemiological studies that target preventable risk factors. For example, a significant number of studies have examined possible dementia risk factors using magnetic resonance imaging (MRI) to detect focal signal abnormalities. However, this method has been mostly applied for diagnosing AD at the dementia stage and it is less effective in detecting the early stages of cognitive impairment and dementia (Hojjati et al., 2018). Several other potential predictors have been utilized to examine the risk of cognitive impairment and dementia. These predictors include the presence of apolipoprotein E (APOE ε4), the Mini-Mental State Examination score, the AD assessment scale-cognitive subscale (ADAS-cog), and the functional assessment questionnaire (FAQ) score (Weiner et al., 2010; Landau et al., 2011; Lee et al., 2014; Woolf et al., 2016; Kueper et al., 2018; Fayosse et al., 2020; Arevalo-Rodriguez et al., 2021; Chen et al., 2022). Unfortunately, while various studies were conducted on independent cohorts, the generalizability of their findings has been limited by small sample sizes (Woolf et al., 2016; Kueper et al., 2018; Arevalo-Rodriguez et al., 2021; Chen et al., 2022). Several studies have found a positive relationship between midlife vascular risk factors (i.e., high blood pressure (HBP), dyslipidemia, and hyperglycemia) and the risk of cognitive impairment and AD/ADRD (Zlokovic et al., 2020; Adkins-Jackson and Belsky, 2022). The potential pathophysiology of this association is supported by findings, suggesting that increased blood pressure, blood dyslipidemia, and hyperglycemia in midlife may trigger and perpetuate chronic brain inflammation. This aspect, in turn, could heighten the risk of brain amyloid β and tau pathology, ultimately leading to an elevated risk of AD and dementia (Pietrzik and Jaeger, 2008; Taguchi, 2009; Waldstein and Wendell, 2010; Correia et al., 2012; Nägga et al., 2018; Fayosse et al., 2020). A few studies examined the associations between cognitive function and AD/ADRD with inflammatory markers (assessed using serum CRP, fibrinogen, interleukin-6 (IL-6), and homocysteine), D-dimer (a marker of fibrinolysis), factor VIII (related to arterial thrombosis) (Carcaillon et al., 2009; Yan et al., 2010; Rubio-Perez and Morillas-Ruiz, 2012; Simon et al., 2018; Lauriola et al., 2021), and the albumin-to- creatinine ratio (a marker of kidney function) (Bikbov et al., 2022). Nevertheless, several research gaps persist: (1) Inconsistent findings have been observed from previous studies, potentially due to the heterogeneous nature of study samples across different studies (Brainerd et al., 2013; Gupta et al., 2019; Liu et al., 2021b). (2) Limited biomarkers were included in previous studies, leading to biases stemming from missed opportunities to assess important biomarkers. (3) There is a scarcity of data from large-scale epidemiological longitudinal studies involving diverse ethnic populations. Our research aims to address this gap by analyzing data from the Multiethnic Study of Atherosclerosis (MESA). Our central research question is whether metabolic disorders, as assessed by the five components of metabolic syndrome (MetSyn) (waist circumference (WC), HBP, elevated glucose and triglyceride (TG) concentrations, and decreased HDL-C concentrations) and eight other biomarkers measured from blood and urine samples, are significantly associated with the risk of cognitive decline, incident AD/ADRD, and AD/ADRD-related mortality. We focused on the most measurable factors typically encountered in primary healthcare settings to examine their association between cognitive decline and the risk of AD/ADRD. The findings from our research are expected not only to underscore the importance of addressing multiple preventable and treatable risk factors in controlling cognitive decline and AD/ADRD at the population level but also to pave the way for further etiological studies. These insights will contribute to the development of more robust risk prediction models and further our understanding of the risk of AD/ADRD.

## Methods

### Study design and study population

MESA is an ongoing cohort study that begun in 2000, investigating the characteristics of subclinical atherosclerosis and the determinants of cardiovascular diseases (CVDs). Its design has been described previously (Center MC, 2001; Bild et al., 2002; Liu et al., 2009). In brief, the MESA cohort comprises a population-based sample of 6,814 men and women aged 45–84 years at baseline. All participants were free of clinical CVD at baseline and were recruited from six US communities (Forsyth County, NC; Northern Manhattan and the Bronx, NY; Baltimore City and Baltimore County, MD; St. Paul, MN; Chicago, IL; and Los Angeles, CA) (Bild et al., 2002; NHLBI BioLINCC, 2022). The MESA cohort participants were 38% white, 28% African American, 22% Hispanic, and 12% Chinese. People with a history of physician-diagnosed myocardial infarction, angina, heart failure, stroke, or transient ischemic attack, or who had undergone an invasive procedure for CVD (coronary artery bypass graft surgery, angioplasty, valve replacement, pacemaker placement, or other vascular surgeries), were excluded from this study (Bild et al., 2002; Yeboah et al., 2011). This study was approved by the institutional review boards of all collaborating institutions and the National Heart, Lung, and Blood Institute (NHLBI), and all participants provided signed informed consent (Yoneyama et al., 2012). Since MESA started in 2000, six repeated examinations (exams 1–6) have been conducted from 2000 to 2018. In our study, we analyzed MESA exams 1–5 because exam 6 was not ready and was not released by the NHLBI for analysis when we developed our study. We obtained the de-identified MESA data from the NHLBI Biologic Specimen and Data Repository Information Coordinating Center (NHLBI-BioLINCC, RMDA V02 1d20120806). We obtained approval from Drexel University Institutional Review Board (#2208009381 and #2308010042). MESA exams 1–5 were conducted from July 2000 to August 2002 (baseline, exam 1), September 2002 to February 2004 (exam 2), March 2004 to September 2005 (exam 3), September 2005 to Mach 2007 (exam 4), and April 2010 to December 2012 (exam 5), respectively. Combining exams 1–5 provides follow-up data through 31 December 2012 for cardiovascular and non-cardiovascular events and through 31 December 2015 for cause-specific mortality. Out of the 6,814 participants included at baseline, we excluded 346 who had missing values for the measures of the five components of MetSyn (WC, HBP, elevated serum glucose, TG concentrations, and decreased HDL-C concentrations) and eight biomarkers [serum C-reactive protein (CRP), fibrinogen, IL-6, D-dimer, homocysteine concentrations, carotid intimal-medial thickness (CIMT), and urine albumin-to-creatinine ratio (ACR)]. We also excluded eight participants who had a clinical diagnosis of AD (assessed by taking medication for AD) and also 20 participants who had not participated in the follow-up or had missed follow-up days. Our final analyses included 6,440 participants (3,040 men and 3,400 women, representing 95% of the original cohort participants).

Assessment of exposures: Body mass index (BMI, kg/m^2^) is calculated as weight (kg) divided by height squared (meters). WC was measured using a standard flexible, tension-regulated tape measure. Systolic/diastolic blood pressure (SBP/DBP) was measured using an automated monitor following a 5-min rest period, with the last two out of three readings averaged and recorded. At each clinic setting, fasting (8–12 h) blood samples were collected from participants and shipped to the MESA central laboratory to measure all the blood factors using standardized protocols (Center MC, 2001; Bild et al., 2002). Total cholesterol and HDL-C were measured from blood samples obtained following a 12-h fast. Low-density lipoprotein cholesterol levels were estimated using the Friedewald equation (Friedewald et al., 1972). Fasting blood glucose (serum) levels were measured using the glucose oxidase method on the Vitros analyzer (Johnson & Johnson Clinical Diagnostics, Rochester, New York) (Yeboah et al., 2011). To define MetSyn, the five MetSyn component cutoff values utilize the modified criteria developed by the American Heart Association, the American Diabetes Association, and the Adults Treatment Panel (ATP) III (Grundy, 2005; Aronow, 2006; Liu et al., 2009, 2014; Inzucchi et al., 2015; American Diabetes Association, 2016). Individuals with MetSyn were classified based on the presence of three or more of the five components: (1) large WC: WC >102 cm in male participants and > 88 cm in female participants, or BMI ≥ 30 kg/m^2^; (2) elevated BP: SBP ≥130 or DBP ≥85 mmHg or anti-hypertensive medication use; (3) elevated TG ≥150 mg/dL; (4) elevated glucose: fasting glucose ≥100 mg/dL or use of glucose-lowering medications; and (5) low level of HDL <40 mg/dL in male participants and HDL <50 mg/dL in female participants.

We further examined the associations by including a group of biomarkers in our analysis. These biomarkers were selected because they have been considered emerging or potential risk factors for AD/ADRD. In the study, we included following eight biomarkers: (1) CRP (a marker of inflammation) measured by a high-sensitivity assay (N-high-sensitivity CRP), (2) fibrinogen (a marker of inflammation, which also plays a critical role in the hemostatic process) measured using immunoprecipitation of fibrinogen antigen using the BNII nephelometer (Dade Behring Inc., Deerfield, Illinois) (Yan et al., 2010), (3) interleukin-6 (an inflammatory interleukin and a marker of immune system activation) measured using ultra-sensitive ELISA (Quantikine HS Human IL-6 Immunoassay; R&D Systems, Minneapolis MN) (Simon et al., 2018), (4) Factor VIII (high factor VIII concentrations are associated with arterial thrombosis) measured utilizing the Sta-R analyzer (STA-Deficient VIII; Diagnostica Stago, Parsippany, NJ), (5) D-dimer (a marker of fibrinolysis and fibrin turnover) measured using an immunoturbidimetric method on the Sta-R analyzer (Liatest D-DI; Diagnostica Stago) (Folsom et al., 2009), (6) homocysteine (a marker of inflammation and vitamin B12 and folate status) measured using high-performance liquid chromatography (Karger et al., 2020), (7) common CIMT (a marker of structural and functional vessel wall properties) measured using B-model ultrasonography (O'Leary et al., 1991), and (8) urine ACR measured using the Vitros 950IRC instrument (Johnson & Johnson Clinical Diagnostics Inc.) (Yu et al., 2011). To have a consistent analysis approach with the five dichotomized MetSyn components, we categorized the other eight markers as binary variables. Elevated CRP was defined as those with CRP ≥3 mg/L and elevated homocysteine ≥12 μmol/L on the basis of previous studies (Oda et al., 2006; Castañon et al., 2007). The remaining six biomarkers were classified according to their 75th or higher than 75th percentile cutoffs (specifically, quartile 4): fibrinogen ≥384 (mg/mL), IL-6 ≥ 1.76 (pg/mL), factor VIII ≥ 199 (%), D-dimer ≥0.34 (μg/mL), CIMT score ≥ 0.95 (mm), and urinary albumin-to-creatinine ratio ≥ 10.0 (mg/g).

Outcomes: Three groups of outcomes were included in the study: (1) Cognitive decline: Cognitive function was evaluated during the fifth MESA follow-up (2010–2012), using the Cognitive Abilities Screening Instrument (CASI, version 2). It should be noted that to assess cognitive impairment and dementia, various instruments have been utilized. The CASI is one of the most commonly used tools to assess overall cognitive function in people at the risk of dementia. The CASI was designed based on symptoms diagnosed as dementia and three cognitive screening tools, such as the Mini-Mental State Examination, the Modified Mini-Mental State Test, and the Hasegawa Dementia Screening Scale. The CASI offers two significant advantages: first, it evaluates overall cognitive function with nine dimensions, providing comprehensive cognitive portraits and second, it demonstrates cross-cultural application in measuring global cognitive function and the risk of dementia (Teng et al., 1994; Fitzpatrick et al., 2015; Chiu et al., 2021). In brief, the CASI includes 25 items representing 9 cognitive domains: attention, concentration, orientation, language, verbal fluency, visual construction, abstraction/judgment, and short- and long-term memory. The CASI score ranges from 0 to 100, with a lower score indicating worse performance (Teng et al., 1994). In the study, we classified cognitive decline as individuals with a CASI score in the lowest 25th percentile of the score distribution. In MESA exam 5, participants with a history of AD/ADRD were excluded while measuring cognitive function. It should be noted that there was no cognitive function evaluation prior to MESA exam 5. Therefore, incident cognitive decline cannot be determined in the study. Out of 4,493 participants who returned for exam 5 in the study sample (i.e., participants without missing values of the study exposures), 4,379 participants completed the CASI test (97% of those returning participants). (2) AD/ADRD: In the MESA study, all cognitive and clinical data were assessed by a convened consensus conference of clinicians (e.g., neurologists, neuropsychologists, and geriatric psychiatrists, geriatricians) experienced in the adjudication of AD/ADRD. The National Institute on Aging (NIA)—Alzheimer’s Association criteria were used to identify AD/ADRD (Albert et al., 2011; McKhann et al., 2011; Hirsch et al., 2022). Hospitalized patients with AD/ADRD were classified using International Classification of Diseases (ICD) codes (ICD-9: 290, 290.1, 290.10-13, 290.2, 290.20-21, 2,903, 2,904, 290.40-43, 290.8-9, 293, 294.1, 331.0, and 331.1). We further classified incident AD for patients who reported taking acetylcholine esterase inhibitors for AD treatment (with the exclusion of baseline AD). (3) Mortality: AD/ADRD-associated death was classified in individuals with a history of incident AD/ADRD or cognitive decline.

Covariates: Several demographic, socioeconomic, and lifestyle factors that were measured in MESA exam 1 (baseline survey) were included in the analysis: age, sex, race/ethnicity, education (an indicator of socioeconomic status), smoking, physical activity, and alcohol consumption status. Race/ethnicity was categorized as white, black, or African American, Hispanic/Latino, and Chinese-American. Education was grouped as ≤ high school, some college or associate degree, and completed college or higher. Smoking was categorized as never, former, and current smokers (Bild et al., 2002). Physical activity was categorized into two groups (regular and non-regular activity). Alcohol consumption was categorized into three groups: (1) never: for those who answered “No” to the question “have you ever consumed alcoholic beverage?”; (2) former: for those who answered “Yes” to the question, “have you ever consumed alcoholic beverage?” and who does not presently drink alcoholic beverages; and (3) current drinkers: for those who reported “they presently drink alcoholic beverages.”

### Statistical analysis

A series of analyses were conducted. First, we described the baseline characteristics of participants based on their incident AD/ADRD status. We used Student’s *t*-tests to examine differences in continuous variables and chi-squared tests in categorical variables. Second, we tested the cross-association of MetSyn and its components, and other elevated biomarkers associated with cognitive decline (defined as low cognitive function, not a change—specifically, those with a CASI score in the lowest 25th percentile at the measures of MESA exam 5) using the logistic regression analysis. Third, we estimated the hazard ratios (HRs) of MetSyn, its components, and other eight markers for the risk of incident AD/ADRD and mortality in individuals with incident AD/ADRD or cognitive decline using Cox proportional hazard (PH) regression models. We examined the Cox PH assumption using a transformation of the Schoenfeld residuals known as the empirical score process, performed via the SAS Proc PHREG/assess PH/resample approach.

All data analyses were conducted using SAS 9.4/STAT 14.2 (SAS Institute Inc., Cary, NC, United States) (SAS Institute Inc., 2014). The reported *p*-values are two-sided, and the significance level was set at 0.05.

## Results

### Baseline characteristics of the study participants by incident AD/ADRD status

Table 1 shows that subjects with incident AD/ADRD had a significantly higher mean age than those without AD/ADRD (73.8 vs. 61.8 years older, *p* < 0.001). Subjects with incident AD/ADRD had significantly lower mean CASI scores, lower mean BMI, higher systolic blood pressure (SBP), and higher glucose concentrations than those without incident AD/ADRD. Among the categorical factors, subjects with incident AD/ADRD had a higher proportion of those with lower education attainment (less than high school), a higher proportion of those with never drinking, and a higher proportion of elevated fibrinogen, factor VIII, D-dimer, homocysteine concentrations, CIMT score, and ACR than those without AD/ADRD.

**Table 1:** Baseline characteristics of the participants by incident Alzheimer’s disease (AD) and AD-related dementia (ADRD).

	Non-AD/ADRD (*n* = 6,298)		AD/ADRD (*n* = 142)		
	Mean or no.	SD or %	No, mean	%, SD	*p*-value
**Continuous variables, mean, SD**					
Age, years	61.8	10.1	73.8	6.4	**<0.001**
CASI, cognitive score*	87.1	11.2	70.4	22.3	**<0.001**
Body mass index, kg/m^2^	28.3	5.4	27.0	5.0	**0.004**
Waist circumference, cm	98.0	14.3	97.3	13.3	0.59
Systolic BP, mm Hg	126.2	21.3	136.5	25.1	**<0.001**
Diastolic BP mm Hg	71.9	10.3	72.2	10.6	0.70
Triglyceride, mg/dL	50.9	14.7	52.5	15.5	0.20
HDL-C, mg/dL	131.6	87.0	130.2	94.7	0.85
Glucose, mg/dL	97.2	30.2	103.4	35.3	**0.016**
**Categorical var., no, %**					
MetSyn, yes	2259	35.9	52	36.6	0.85
Sex, males	2964	47.1	76	53.5	0.12
Race/ethnicity					0.87
White	2449	38.9	58	40.8	
Chinese	769	12.2	15	10.6	
African American	1699	27.0	37	26.1	
Hispanics	1381	21.9	32	22.5	
Education					**0.002**
<High school	1116	17.8	42	29.6	
High school	1140	18.2	25	17.6	
Some college	1781	28.4	32	22.5	
College and higher	2242	35.7	43	30.3	
Smoking					0.15
Never smoked	4781	75.9	114	80.3	
Ex-smokers	561	8.9	13	9.2	
Current smokers	956	15.2	15	10.6	
Physical activity					
Regular	1582	25.2	27	19.0	0.09
Alcohol consumption					**0.005**
Never use	1277	20.3	41	28.9	
Former use	1505	24.0	37	26.1	
Current use	3498	55.7	64	45.1	
**Elevated biomarkers**					
CRP, mg/L	2240	35.6	42	29.6	0.14
Fibrinogen, mg/dL	1568	24.9	46	32.4	**0.042**
Interleukin-6, pg/mL	1572	25.0	36	25.4	0.92
Factor VIII, %	1590	25.2	49	34.5	**0.01**
D-dimer (μg/mL)	1567	24.9	69	48.6	**<0.001**
Homocysteine, μmol/L	838	13.3	45	31.7	**<0.001**
CIMT, mm	1604	25.5	69	48.6	**<0.001**
Album/Cre ratio, mg/g	1559	24.8	58	40.8	**<0.001**

AD/ADRD, Alzheimer's disease/AD-related dementia; CASI, Cognitive Abilities Screening Instrument, measured in MESA exam 5 (*n* = 4,379); HDL-C, High-density lipoprotein cholesterol; CRP, C-reactive protein; CIMT, Carotid intimal-medial thickness; Album/Cre ratio, urinary albumin-to-creatinine ratio. See text for details of biomarkers cutoff values. Significant difference: *p* < 0.05 in bold.

### Cross-sectional association between risk factors and cognitive decline

**Figure 1:**
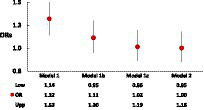
Changes in ORs of MetSyn associated with cognitive decline, when adjusting for age–sex (Model 1), plus race (Model 1b), plus education (Model 1c), and full-adjusted (Model 2).

Table 2 shows that after adjustment for age and sex (Model 1), MetSyn was significantly associated with the odds of cognitive decline (OR = 1.32, 95%CI: 1.14–1.53). Among the individual factors, HBP, elevated TG, low high-density lipoprotein (HDL), elevated glucose, fibrinogen, factor VIII, D-dimer, and homocysteine concentrations, as well as ACR were significantly associated with cognitive decline (Model 1). However, after further adjustment by including race/ethnicity, education, and lifestyle factors (Model 2, the full-adjusted model), this MetSyn–cognitive decline association became non-significant (OR = 1.00, 95% CI: 0.85–1.18). Similar to this observation, Model 2 indicated that only elevated homocysteine concentrations and ACR remained significantly associated with increased odds of cognitive decline (OR = 1.19, 95%CI: 1.06–1.69 for homocysteine, and OR = 1.24, 95% CI: 1.03–1.48 for ACR). Given this significant change in the ORs of MetSyn associated with cognitive decline from Model 1 to Model 2, we further investigated the main factors contributing to this change by conducting two subset analyses with adjusting for race/ethnicity and education in a step-by-step entry approach in Model 1b and Model 1c. The results demonstrated that after adjusting for age, sex, and race/ethnicity (Model 1b of Figure 1 and Supplementary Table S1), the OR (95% CI) of MetSyn-associated cognitive decline attenuated to 1.11 (0.95–1.30, *p* = 0.18), indicating a 64.9% of the OR reduction from Model 1 to Model 1b [estimated by (OR1-OR2)/(OR1-1)*100]. After further adjustment for education, the ORs attenuated to 1.02 (95% CI: 0.86–1.19, *p* = 0.86, Model 1c of Figure 1 and Supplementary Table S1). Among these adjusted covariates, the results show that age, race/ethnicity, education, and alcohol consumption status are significantly associated with cognitive decline (Supplementary Table S1).

**Table 2:** Adjusted odds ratios [ORs, 95% confidence intervals (CIs)] for cognitive decline associated with metabolic syndrome, its components, and multiple biomarkers.

	Model 1			Model 2		
	OR	(95% CI)	*p*-value	OR	(95% CI)	*p*-value
MetSyn (yes vs. no)	1.32	(1.14–1.53)	**<0.001**	1.00	(0.85–1.18)	0.99
**MetSyn components**						
Large WC	1.07	(0.93–1.24)	0.34	0.87	(0.74–1.03)	0.11
HBP	1.35	(1.17–1.56)	**<0.001**	1.14	(0.97–1.34)	0.12
Elevated TG	1.19	(1.02–1.38)	**0.026**	0.99	(0.83–1.17)	0.86
Low HDL-C	1.24	(1.07–1.44)	**0.004**	0.97	(0.83–1.15)	0.75
Elevated Glucose	1.58	(1.35–1.86)	**<0.001**	1.13	(0.95–1.35)	0.18
**Elevated biomarkers**						
C-reactive protein	0.98	(0.83–1.16)	0.81	0.84	(0.75–1.05)	0.16
Fibrinogen	1.19	(1.01–1.40)	**0.043**	0.94	(0.79–1.13)	0.51
Interleukin-6	1.08	(0.91–1.28)	0.36	0.92	(0.77–1.11)	0.39
Factor VIII	1.20	(1.02–1.41)	**0.028**	1.16	(0.97–1.39)	0.11
D-dimer	1.25	(1.06–1.47)	**0.009**	1.13	(0.94–1.35)	0.20
Homocysteine	1.22	(1.03–1.44)	**0.019**	1.19	(1.06–1.69)	**0.019**
CIMT	1.11	(0.93–1.31)	0.25	1.08	(0.89–1.30)	0.45
Album/Cre ratio	1.47	(1.24–1.73)	**<0.001**	1.24	(1.03–1.48)	**0.021**

MetSyn, Cardiometabolic syndrome; WC, Waist circumference; HBP, High blood pressure; TG, triglyceride; HDL-C, high-density lipoprotein cholesterol; CIMT, carotid intimal-medial thickness; Album/Cre, urine albumin-to-creatinine ratio; Model 1, Adjusted age and sex; Model 2, Adjusted for age, sex, race, education, smoking, physical activity, and alcohol consumption. Significant difference: *p* < 0.05 in bold.

### Longitudinal association of MetSyn and biomarkers with risk of incident ADRD

Among 6,440 participants at baseline, followed-up by the end of 2015 (a total of 45,608 person-years follow-up), 142 participants were classified as incident AD/ADRD. Table 3 shows that, after adjustment for age and sex (Model 1) and a full adjustment for multiple covariates (Model 2), baseline MetSyn and its components were not independently associated with the risk of incident AD/ADRD. Elevated blood D-dimer concentrations had a borderline significance for the risk of incent AD/ADRD (*p* = 0.048). Elevated homocysteine concentrations and urine ACR were significantly associated with the risk of incident AD/ADRD (*p* = 0.005 in elevated homocysteine concentrations and *p* = 0.038 in elevated ACR).

**Table 3:** Adjusted hazard ratios (HRs, 95%CI) of MetSyn, its components, and biomarkers associated with incident AD/ADRD.

	Model 1			Model 2		
Case/person-yrs.	142/45,608 person-yrs.			142/45,608 person-yrs.		
HRs of risk factors	HR	(95%CI)	*p*-value	HR	(95%CI)	*p*-value
MetSyn (yes vs. no)	0.99	(0.70–1.41)	0.96	0.94	(0.66–1.33)	0.72
**MetSyn components**						
Large WC	0.85	(0.60–1.21)	0.37	0.82	(0.57–1.16)	0.25
HBP	1.13	(0.77–1.66)	0.52	1.10	(0.75–1.61)	0.63
Elevated TG	0.97	(0.67–1.40)	0.87	0.95	(0.65–1.38)	0.77
Low HDL-C	0.95	(0.66–1.36)	0.78	0.89	(0.61–1.29)	0.53
Elevated glucose	1.27	(0.90–1.81)	0.17	1.21	(0.85–1.73)	0.30
**Elevated biomarkers**						
C-reactive protein	0.85	(0.59–1.23)	0.39	0.83	(0.57–1.22)	0.34
Fibrinogen	1.20	(0.84–1.73)	0.31	1.16	(0.81–1.67)	0.41
Interleukin-6	0.86	(0.59–1.26)	0.45	0.84	(0.58–1.22)	0.36
Factor VIII	1.23	(0.86–1.75)	0.26	1.23	(0.85–1.76)	0.27
D-dimer	1.42	(1.01–1.98)	**0.043**	1.41	(1.00–1.99)	**0.048**
Homocysteine	1.79	(1.21–2.65)	**0.004**	1.76	(1.18–2.60)	**0.005**
CIMT	1.18	(0.84–1.67)	0.34	1.19	(0.84–1.68)	0.32
Album/Cre ratio	1.48	(1.05–2.08)	**0.025**	1.44	(1.02–2.03)	**0.038**

HR, Hazard ratios are estimated using Cox's models to test time-to-event risk; WC, Waist circumference; HBP, High blood pressure; TG, Triglyceride; HDL-C, High-density lipoprotein cholesterol; CIMT, Carotid intimal-medial thickness; Album/Cre ratio, Urine albumin-to-creatinine ratio; Model 1, Adjusted age and sex; Model 2, Adjusted for age, sex, race, education, smoking, physical activity, and alcohol consumption. Significant difference: *p* < 0.05 in bold.

### Longitudinal association of MetSyn and biomarkers with all-cause mortality in those with incident AD/ADRD or cognitive decline

**Figure 2:**
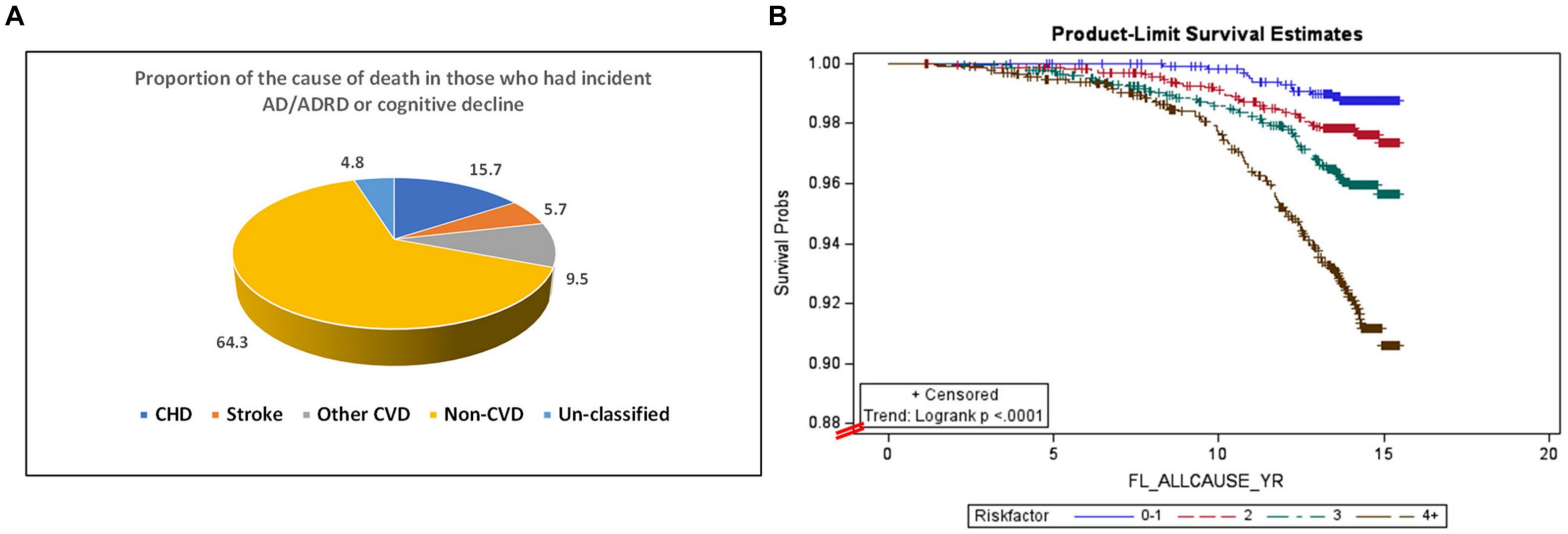
Percent of all-cause mortality from cardiovascular disease (total CVD: 30.9%, of them, CHD: 15.7%, stroke: 5.7%, and other CVD: 9.5%), and non-CVD (64.3%) in those with AD/ADRD or cognitive decline **(A)**, and the association between an increased number of exposures to the risk factors and all-cause mortality in those with AD/ADRD or cognitive decline **(B)**.

Among 6,440 participants in MESA exam 1, 5448 were followed-up by the end of 2015 (a total of 83,942 person-years follow-up), with valid follow-up information. 210 all-cause deaths were observed. Within this group, 83 of the deaths were among the 142 individuals who had incident AD/ADRD (a death rate of 58.5%). The remaining 127 deaths were among the 1,066 individuals who had cognitive decline but had not yet developed AD/ADRD (a death rate of 11.9%). Table 4 shows that after adjustment for multiple covariates (Model 2), HBP, elevated glucose, CRP, IL-6, D-dimer, and homocysteine concentrations, as well as urine ACR were significantly associated with all-cause mortality in those with AD/ADRD or cognitive decline (*p* < 0.05 or *p* < 0.001). Figure 2A shows that among the 210 all-cause deaths who had a history of AD/ADRD or cognitive decline, 31% of them died from CVD (15.7% from coronary heart disease, 5.7% from stroke, and 9.5% from other forms of heart disease), and 64.3% from non- CVD. Figure 2B depicts the risk trend of exposures to an increased number of the study risk factors associated with all-cause mortality in those with AD/ADRD or cognitive decline compared to their corresponding counterparts.

**Table 4:** Adjusted hazard ratios (HRs, 95% confidence interval) of metabolic syndrome, its components, and biomarkers associated with all-cause mortality in those with AD/ADRD or cognitive decline.

	Model 1			Model 2		
Case/person-yrs.	210/83,942 person-years			210/83,942 person-years		
Risk factors for ADRD death	HR	(95% CI)	*p*-value	HR	(95% CI)	*p*-value
MetSyn (yes vs. no)	1.27	(0.96–1.68)	0.10	1.13	(0.85–1.50)	0.40
**MetSyn components**						
Large WC	1.17	(0.88–1.54)	0.28	1.07	(0.81–1.42)	0.64
High blood pressure	1.78	(1.26–2.52)	**0.001**	1.70	(1.20–2.40)	**0.003**
Elevated TG	0.96	(0.71–1.31)	0.82	0.92	(0.67–1.24)	0.57
Low HDL-C	1.15	(0.86–1.54)	0.34	1.06	(0.79–1.41)	0.72
Elevated glucose	1.58	(1.19–2.10)	**0.002**	1.41	(1.06–1.89)	**0.02**
**Elevated biomarkers**						
C-reactive protein	1.54	(1.16–2.05)	**0.003**	1.42	(1.06–1.88)	**0.017**
Fibrinogen	1.31	(0.97–1.77)	0.08	1.26	(0.93–1.71)	0.14
Interleukin-6	1.78	(1.34–2.36)	**<0.001**	1.66	(1.25–2.20)	**0.001**
Factor VIII	1.06	(0.78–1.43)	0.72	1.05	(0.77–1.43)	0.77
D-dimer	1.59	(1.20–2.11)	**0.001**	1.54	(1.16–2.05)	**0.003**
homocysteine	2.00	(1.46–2.72)	**<0.001**	1.89	(1.38–2.58)	**<0.001**
CIMT	1.14	(0.85–1.52)	0.38	1.11	(0.83–1.48)	0.47
Album/Cre ratio	2.21	(1.67–2.93)	**<0.001**	2.08	(1.57–2.76)	**<0.001**

HR, Hazard ratios are estimated using Cox's models to test time-to-event risk; WC, Waist circumference; HBP, High blood pressure; TG, Triglyceride; HDL-C, High-density lipoprotein cholesterol; CIMT, Carotid intimal-medial thickness; Album/Cre ratio, Urine albumin-to-creatinine ratio; Model 1, Adjusted age and sex; Model 2, Adjusted for age, sex, race, education, smoking, physical activity, and alcohol consumption. Significant difference: *p* < 0.05 in bold.

## Discussion

AD and ADRD have emerged as significant public health concerns in the USA and globally. Historically, AD/ADRD was predominantly approached and studied within the realm of genetics. However, recent research, including our earlier reports, has revealed several potential factors that are both preventable and treatable, such as HBP (Liu et al., 2022), dyslipidemia, dysglycemia (Liu et al., 2021a,c), elevated fibrinogen (Lauriola et al., 2021), and elevated homocysteine (Kim et al., 2019), that contribute to the development of AD/ADRD. In the present study, we explored the association between various risk factors and the risk of AD/ADRD mortality over an average follow-up period of 13 years. Among 13 factors included in this study, HBP, elevated glucose, CRP, homocysteine, IL-6, and elevated urine ACR were significantly associated with the risk of all-cause mortality in those with incident AD/ADRD or cognitive decline. Meanwhile, our results indicate that elevated D-dimer and homocysteine concentrations and ACR were significantly associated with incident AD/ADRD. Elevated homocysteine concentrations and ACR were also significantly associated with cognitive decline. Additionally, our research unveiled a dose–response association, demonstrating that an increased number of exposures to these risk factors corresponded to a higher risk of all-cause mortality in those with AD/ADRD or cognitive decline. Our comprehensive analyses shed light on the intricate web of factors influencing AD/ADRD and underscore the importance of addressing preventable and modifiable elements in the prevention and treatment of these conditions.

In our study, we conducted a cross-sectional analysis to examine the association between MetSyn and biomarkers with the risk of cognitive decline. This approach was necessitated by the absence of a baseline measure of cognitive function prior to MESA exam 5. Our findings, as shown in the age–sex-adjusted model (Model 1 of Table 2), demonstrated a significant association between MetSyn, several MetSyn components, and biomarkers with the risk of cognitive decline. However, many of these associations lost significance after further adjustment for race/ethnicity, education, and lifestyle factors (smoking, physical activity, and alcohol consumption). These results indicate that multiple variables, particularly those related to demographics (age, sex, and race/ethnicity), education, and lifestyle factors, play a strong role in influencing the association between MetSyn and cognitive decline. In our previous studies, we have demonstrated that race/ethnicity, education, and lifestyle-related factors (i.e., obesity and smoking) are significantly associated with the risk of MetSyn (Liu et al., 2012, 2014, 2020). Given that our current study focuses on investigating the associations of metabolic disorders and biomarkers with the risk of cognitive decline and AD/ADRD, we incorporated these covariates (demographic, education, and lifestyle factors) as confounders instead of predictors in the analyses. It is essential to note that, even after adjusting for demographics, socioeconomic status (assessed by educational level), and lifestyle factors, elevated homocysteine concentrations and ACR remained independently and significantly associated with the risk of cognitive impairment.

Several studies have examined the relationship between elevated blood homocysteine concentrations and the risk of cognitive decline, with varying results. While some researchers have reported a significant association (Smith and Refsum, 2016; Kim et al., 2019; Lauriola et al., 2021), not all have yielded the same conclusions (Reitz et al., 2009). For example, Lauriola et al. conducted a study involving 929 participants aged 60–93 years, including individuals with mild cognitive impairment (MCI, *n* = 126) and those without MCI (*n* = 803). Their findings revealed a significant association between elevated homocysteine concentrations and increased odds of MCI, possible AD, and vascular AD (*p* < 0.01) (Lauriola et al., 2021). In contrast, Reitz et al. analyzed a cohort sample of 516 participants, with a mean age of 77 years, who did not have MCI or dementia at baseline. Over a 5.2-year follow-up period, the researchers found no significant association between blood homocysteine concentrations and the risk of MCI (Reitz et al., 2009). These discrepancies in findings may be attributed to differences in study populations or the relatively small sample size in the study conducted by Reitz et al. Nevertheless, the inconsistent results emphasize the need for further in-depth investigations into these associations.

The association between an elevated ACR and the risk of cognitive decline and AD/ADRD has also been observed by others (Bikbov et al., 2022). Although the precise mechanisms by which elevated homocysteine concentrations and ACR may contribute to the development of cognitive decline and AD/ADRD remain incompletely understood, there are several potential risk pathways to consider. These may include HBP, inflammation, and kidney dysfunction (assessed by ACR), all of which could lead to brain hypoxia, endothelial damage, and injuries (Rubio-Perez and Morillas-Ruiz, 2012; Smith and Refsum, 2016; Kim et al., 2019; Lauriola et al., 2021; Bikbov et al., 2022). Additionally, increased homocysteine concentrations serve as markers of impairment in vitamin B_12_ and folate metabolism, which may result in neuronal injury and an increased risk of cognitive decline and AD/ADRD (Moretti et al., 2017; Loures et al., 2019).

In the context of AD/ADRD research, substantial evidence underscores the role of amyloid-β (Aβ) deposition in the brain as the initiating factor in the pathogenesis of AD/ADRD. However, it is becoming increasingly clear that, alongside abnormal amyloid metabolism, other pathophysiological mechanisms are likely at play. Notably, hemostatic abnormalities and oxidative stress are emerging as potential contributors to the pathophysiological process associated with AD/ADRD (Rubio-Perez and Morillas-Ruiz, 2012; Loures et al., 2019). In our retrospective cohort analyses, we observed that elevated D-dimer concentrations, which are markers of hemostatic abnormalities, were significantly associated with AD/ADRD. While hypercoagulability promotes fibrin formation, it also heightens the risk of thrombosis. It is speculated that hemostatic abnormalities may predispose individuals to the development of microthrombi, which, in turn, can lead to compromised perfusion within the cerebral microcirculation. This aspect, in all likelihood, contributes to the impairment of cognitive function and other neurological processes (Carcaillon et al., 2009; Loures et al., 2019). It is important to note that, due to the nature of a large population-based MESA study, other directly AD/ADRD-related measurements obtained from samples of cerebrospinal fluid (surrounding the brain) were not available, such as tau and Aβ concentrations, nor tau neurofibrillary tangles and Aβ plaques (using positron emission tomography scans). Therefore, we are unable to test the associations of the study markers with tau and Aβ concentrations. Nevertheless, our findings emphasize the significance of further etiological investigations that consider potential pathways involving hemostatic abnormalities in the context of AD/ADRD risk.

Our current study offers several advantages. First, the MESA dataset stands out as one of the few studies that includes diverse study populations, including white, black, Hispanic, and Chinese participants. Second, the meticulous measurements of multiple factors in the study were conducted using standardized approaches, and biomarkers were centrally measured in one coordinating laboratory center within the MESA framework. This approach ensured consistency and accuracy in the study. Third, with a sample size of 6,440 participants, our study ranks among the largest in the existing literature on population-based cohort studies with the inclusion of both genders and diverse populations. Fourth, we employed a rigorous and robust analysis design to investigate both cross-sectional associations (risk factors for the odds of cognitive decline) and longitudinal associations (risk factors for the incidence of AD/ADRD and all-cause mortality in those with AD/ADRD or cognitive decline), while carefully adjusting for multiple covariates.

Nonetheless, it is important to acknowledge several limitations inherent in our analyses. First, association analyses were conducted between risk factors measured at baseline and the study outcomes at exam 5 for cognitive decline, and follow-up for measures of AD/ADRD and mortality through 31 December 2015. Our analyses do not take into account any changes in the baseline risk factors; their values may vary during the follow-up, which may potentially lead to either an over- or underestimation of the study associations. Second, the incident AD/ADRD cases may have been underestimated because of a competing cause of death that may have occurred before an individual had a chance to develop AD/ADRD. Third, given that the MESA study is ongoing, it is possible that some participants may develop AD/ADRD later in life. Therefore, findings from the current analysis may underestimate the association because some outcomes (AD/ADRD) may occur after the conclusion of the current analysis period. Fourth, because cognitive function assessments were lacking at baseline, we analyzed the cross-sectional association of baseline MetSyn and the study biomarkers with the odds of cognitive decline (measured at the MESA exam 5). Consequently, these cross-sectional analyses do not allow for an interpretation of causal associations between the study risk factors and cognitive decline.

## Conclusion

Despite these limitations, the results of this study provide new evidence and highlight the significance of preventable and treatable factors, including HBP, hyperglycemia, elevated CRP, factor VII, D-dimer, homocysteine, and kidney dysfunction, as potential factors for reducing the risk of cognitive decline and AD/ADRD. This research contributes to a growing body of evidence emphasizing the interconnectedness of multiple factors with the risk of cognitive decline and AD/ADRD, which adds further suggestions to healthcare practice in controlling the risk of these conditions. However, further research is essential to explore the mechanistic pathways linking various disorders with cognitive decline and dementia risk, which includes investigating variations in the risk of AD/ADRD by race/ethnicity, sex, and the interrelations of these study factors. Additionally, there is a need to develop prediction models for the early detection of cognitive impairment and dementia risk and to develop targeted strategies for prevention and treatment.

## Data availability statement

The data, analyzed in this study, is subject to the following licenses/restrictions: The data analyzed in this study was from the National Heart, Lung, and Blood Institute (NHLBI) Biologic Specimen and Data Repository Information Coordinating Center (BioLINCC). Investigators who are interested in the data may contact the NHLBI – BioLINCC. Requests to access these datasets should be directed to https://biolincc.nhlbi.nih.gov/home/.

## Ethics statement

The present data analysis project, used unidentifiable / de-identified MESA dataset from the National Heart, Lung, and Blood Institute (NHLBI-BioLINCC). The data analysis project did not use human subject research as defined by DHHS or FDA regulations. Written informed consent for participation was not required from the participants or the participants’ legal guardians/next of kin for this analysis project in accordance with the national legislation and institutional requirements.

## Author contributions

LL: Conceptualization, Data curation, Formal analysis, Funding acquisition, Resources, Validation, Visualization, Writing – original draft, Writing – review & editing. EG: Writing – review & editing. XZ: Writing – review & editing. GG: Writing – review & editing. NM: Writing – review & editing. SV: Writing – review & editing. JS: Writing – review & editing. RD-G: Writing – review & editing. HE: Writing – review & editing.

## References

[ref1] Adkins-Jackson P. B. Belsky D. W. (2022). Alzheimer's disease risk biomarkers: progress and challenges. Lancet Healthy Longev. 3, e575–e576. doi: 10.1016/S2666-7568(22)00191-X 36102768

[ref2] Albert M. S. DeKosky S. T. Dickson D. Dubois B. Feldman H. H. Fox N. C.. (2011). The diagnosis of mild cognitive impairment due to Alzheimer's disease: recommendations from the National Institute on Aging-Alzheimer's Association workgroups on diagnostic guidelines for Alzheimer's disease. Alzheimers Dement. 7, 270–279. doi: 10.1016/j.jalz.2011.03.008, PMID: 21514249 PMC3312027

[ref3] American Diabetes Association (2016). 8. Cardiovascular disease and risk management. Diabetes Care 39, S60–S71. doi: 10.2337/dc16-S011 26696684

[ref4] Arevalo-Rodriguez I. Smailagic N. Roqué-Figuls M. Ciapponi A. Sanchez-Perez E. Giannakou A.. (2021). Mini-mental state examination (MMSE) for the early detection of dementia in people with mild cognitive impairment (MCI). Cochrane Database Syst. Rev. 7:CD010783. doi: 10.1002/14651858.CD010783.pub3 34313331 PMC8406467

[ref5] Aronow W. S. (2006). ACC/AHA guideline update: treatment of heart failure with reduced left ventricular ejection fraction. Geriatrics 61, 22–29. PMID: 16522132

[ref6] Bikbov B. Soler M. J. Pešić V. Capasso G. Unwin R. Endres M.. (2022). Albuminuria as a risk factor for mild cognitive impairment and dementia—what is the evidence? Nephrol. Dial. Transpl. 37, ii55–ii62. doi: 10.1093/ndt/gfab261 PMC8713154 34739540

[ref7] Bild D. E. Bluemke D. A. Burke G. L. Detrano R. Diez Roux A. V. Folsom A. R.. (2002). Multi-ethnic study of atherosclerosis: objectives and design. Am. J. Epidemiol. 156, 871–881. doi: 10.1093/aje/kwf113 12397006

[ref8] Brainerd C. Reyna V. Petersen R. Smith G. Kenney A. Gross C.. (2013). The apolipoprotein E genotype predicts longitudinal transitions to mild cognitive impairment but not to Alzheimer's dementia: findings from a nationally representative study. Neuropsychology 27, 86–94. doi: 10.1037/a0030855, PMID: 23356599 PMC3874553

[ref9] Carcaillon L. Gaussem P. Ducimetiere P. Giroud M. Ritchie K. Dartigues J.. (2009). Elevated plasma fibrin D-dimer as a risk factor for vascular dementia: the Three-City cohort study. J. Thromb. Haemost. 7, 1972–1978. doi: 10.1111/j.1538-7836.2009.03603.x, PMID: 19735443

[ref10] Castañon M. M. Lauricella A. M. Kordich L. Quintana I. (2007). Plasma homocysteine cutoff values for venous thrombosis. Clin. Chem. Lab. Med. 45, 232–236. doi: 10.1515/CCLM.2007.038, PMID: 17311514

[ref11] Center MC. Multi-ethnic study of atherosclerosis field center manual of operations. Seattle, WA: University of Washington. (2001).

[ref12] Chen Y. Qian X. Zhang Y. Su W. Huang Y. Wang X.. (2022). Prediction models for conversion from mild cognitive impairment to Alzheimer’s disease: a systematic review and meta-analysis. Front. Aging Neurosci.:840386:14. doi: 10.3389/fnagi.2022.840386 35493941 PMC9049273

[ref13] Chiu E.-C. Chien T.-H. Lee Y.-C. (2021). Measurement equivalence between the original and estimated mini-mental state examination in people with dementia. Int. J. Environ. Res. Public Health 18:7616. doi: 10.3390/ijerph18147616, PMID: 34300067 PMC8305709

[ref14] Correia S. C. Santos R. X. Cardoso S. Carvalho C. Candeias E. Duarte A. I.. (2012). Alzheimer disease as a vascular disorder: where do mitochondria fit? Exp. Gerontol. 47, 878–886. doi: 10.1016/j.exger.2012.07.006, PMID: 22824543

[ref15] Fayosse A. Nguyen D.-P. Dugravot A. Dumurgier J. Tabak A. G. Kivimäki M.. (2020). Risk prediction models for dementia: role of age and cardiometabolic risk factors. BMC Med. 18, 1–10. doi: 10.1186/s12916-020-01578-x 32423410 PMC7236124

[ref16] Fitzpatrick A. L. Rapp S. R. Luchsinger J. Hill-Briggs F. Alonso A. Gottesman R.. (2015). Sociodemographic correlates of cognition in the multi-ethnic study of atherosclerosis (MESA). Am. J. Geriatr. Psychiatry 23, 684–697. doi: 10.1016/j.jagp.2015.01.003, PMID: 25704999 PMC4465027

[ref17] Folsom A. R. Delaney J. A. Lutsey P. L. Zakai N. A. Jenny N. S. Polak J. F.. (2009). Associations of factor VIIIc, D-dimer, and plasmin–antiplasmin with incident cardiovascular disease and all-cause mortality. Am. J. Hematol. 84, 349–353. doi: 10.1002/ajh.21429, PMID: 19472201 PMC2950108

[ref18] Friedewald W. T. Levy R. I. Fredrickson D. S. (1972). Estimation of the concentration of low-density lipoprotein cholesterol in plasma, without use of the preparative ultracentrifuge. Clin. Chem. 18, 499–502. doi: 10.1093/clinchem/18.6.499, PMID: 4337382

[ref19] Grundy S. M. (2005). A constellation of complications: the metabolic syndrome. Clin. Cornerstone 7, 36–45. doi: 10.1016/S1098-3597(05)80066-3, PMID: 16473259

[ref20] Gupta Y. Lama R. K. Kwon G.-R. Weiner M. W. Aisen P. Weiner M.. (2019). Prediction and classification of Alzheimer’s disease based on combined features from apolipoprotein-E genotype, cerebrospinal fluid, MR, and FDG-PET imaging biomarkers. Front. Comput. Neurosci. 13:72. doi: 10.3389/fncom.2019.00072, PMID: 31680923 PMC6805777

[ref21] Hirsch J. A. Michael Y. L. Moore K. A. Melly S. Hughes T. M. Hayden K.. (2022). Longitudinal neighbourhood determinants with cognitive health and dementia disparities: protocol of the multi-ethnic study of atherosclerosis Neighborhoods and aging prospective cohort study. BMJ Open 12:e066971. doi: 10.1136/bmjopen-2022-066971, PMID: 36368762 PMC9660618

[ref22] Hojjati S. H. Ebrahimzadeh A. Khazaee A. Babajani-Feremi A. AsDN I. (2018). Predicting conversion from MCI to AD by integrating rs-fMRI and structural MRI. Comput. Biol. Med. 102, 30–39. doi: 10.1016/j.compbiomed.2018.09.004, PMID: 30245275

[ref23] Inzucchi S. E. Bergenstal R. M. Buse J. B. Diamant M. Ferrannini E. Nauck M.. (2015). Management of hyperglycemia in type 2 diabetes, 2015: a patient-centered approach: update to a position statement of the American Diabetes Association and the European Association for the Study of diabetes. Diabetes Care 38, 140–149. doi: 10.2337/dc14-2441, PMID: 25538310

[ref24] Karger A. B. Steffen B. T. Nomura S. O. Guan W. Garg P. K. Szklo M.. (2020). Association between homocysteine and vascular calcification incidence, prevalence, and progression in the MESA cohort. J. Am. Heart Assoc. 9:e013934. doi: 10.1161/JAHA.119.013934, PMID: 32013703 PMC7033888

[ref25] Kim S. Choi B. Y. Nam J. H. Kim M. K. Oh D. H. Yang Y. J. (2019). Cognitive impairment is associated with elevated serum homocysteine levels among older adults. Eur. J. Nutr. 58, 399–408. doi: 10.1007/s00394-017-1604-y, PMID: 29322314

[ref26] Kueper J. K. Speechley M. Montero-Odasso M. (2018). The Alzheimer’s disease assessment scale–cognitive subscale (ADAS-cog): modifications and responsiveness in pre-dementia populations. A narrative review. J. Alzheimers Dis. 63, 423–444. doi: 10.3233/JAD-170991, PMID: 29660938 PMC5929311

[ref27] Landau S. M. Harvey D. Madison C. M. Koeppe R. A. Reiman E. M. Foster N. L.. (2011). Associations between cognitive, functional, and FDG-PET measures of decline in AD and MCI. Neurobiol. Aging 32, 1207–1218. doi: 10.1016/j.neurobiolaging.2009.07.002, PMID: 19660834 PMC2891865

[ref28] Lauriola M. D’Onofrio G. Ciccone F. Germano C. Cascavilla L. Paris F.. (2021). Relationship of homocysteine plasma levels with mild cognitive impairment, Alzheimer’s disease, vascular dementia, psychobehavioral, and functional complications. J. Alzheimers Dis. 82, 235–248. doi: 10.3233/JAD-210166, PMID: 34057086 PMC8293649

[ref29] Lee S. J. Ritchie C. S. Yaffe K. Stijacic Cenzer I. Barnes D. E. (2014). A clinical index to predict progression from mild cognitive impairment to dementia due to Alzheimer's disease. PLoS One 9:e113535. doi: 10.1371/journal.pone.0113535, PMID: 25486250 PMC4259326

[ref30] Liu L. Gracely E. J. Yin X. Eisen H. J. (2021a). Impact of diabetes mellitus and cardiometabolic syndrome on the risk of Alzheimer’s disease among postmenopausal women. World J. Diabetes 12, 69–83. doi: 10.4239/wjd.v12.i1.69 33520109 PMC7807256

[ref31] Liu L. Hayden K. M. May N. S. Haring B. Liu Z. Henderson V. W.. (2022). Association between blood pressure levels and cognitive impairment in older women: a prospective analysis of the Women's Health Initiative memory study. Lancet Healthy Longev. 3, e42–e53. doi: 10.1016/S2666-7568(21)00283-X, PMID: 35112096 PMC8804967

[ref32] Liu L. Lima J. A. Post W. S. Szklo M. (2021b). Associations of time-varying obesity and metabolic syndrome with risk of incident heart failure and its subtypes: findings from the multi-ethnic study of atherosclerosis. Int. J. Cardiol. 338, 127–135. doi: 10.1016/j.ijcard.2021.05.051, PMID: 34089770

[ref33] Liu L. Miura K. Fujiyoshi A. Kadota A. Miyagawa N. Nakamura Y.. (2014). Impact of metabolic syndrome on the risk of cardiovascular disease mortality in the United States and in Japan. Am. J. Cardiol. 113, 84–89. doi: 10.1016/j.amjcard.2013.08.042, PMID: 24169008

[ref34] Liu L. Nettleton J. A. Bertoni A. G. Bluemke D. A. Lima J. A. Szklo M. (2009). Dietary pattern, the metabolic syndrome, and left ventricular mass and systolic function: the multi-ethnic study of atherosclerosis. Am. J. Clin. Nutr. 90, 362–368. doi: 10.3945/ajcn.2009.27538, PMID: 19515735 PMC2709312

[ref35] Liu L. Núṅez A. E. An Y. Liu H. Chen M. Ma J.. (2014). Burden of cardiovascular disease among multi-racial and ethnic populations in the United States: an update from the National Health Interview Surveys. Front. Cardiovasc. Med. 1:8. doi: 10.3389/fcvm.2014.00008 26664859 PMC4668845

[ref36] Liu L. Volpe S. L. Ross J. A. Grimm J. A. Van Bockstaele E. J. Eisen H. J. (2021c). Dietary sugar intake and risk of Alzheimer's disease in older women. Nutr. Neurosci. 25, 2302–2313. doi: 10.1080/1028415X.2021.1959099 34328409

[ref37] Liu L. Wang F. Gracely E. J. Moore K. Melly S. Zhang F.. (2020). Burden of uncontrolled hyperglycemia and its association with patients characteristics and socioeconomic status in Philadelphia, USA. Health Equity 4, 525–532. doi: 10.1089/heq.2020.0076, PMID: 34095699 PMC8175259

[ref38] Liu L. Yin X. Morrissey S. (2012). Global variability in diabetes mellitus and its association with body weight and primary healthcare support in 49 low-and middle-income developing countries. Diab. Med. 29, 995–1002. doi: 10.1111/j.1464-5491.2011.03549.x, PMID: 22150805

[ref39] Loures C. M. G. Duarte R. C. F. Silva M. V. F. Cicarini W. B. de Souza L. C. Caramelli P.. (2019). Hemostatic abnormalities in dementia: a systematic review and Meta-analysis. Semin. Thromb. Hemost. 45, 514–522. doi: 10.1055/s-0039-1688444 31096308

[ref40] McKhann G. M. Knopman D. S. Chertkow H. Hyman B. T. Jack C. R. Jr. Kawas C. H.. (2011). The diagnosis of dementia due to Alzheimer’s disease: recommendations from the National Institute on Aging-Alzheimer’s association workgroups on diagnostic guidelines for Alzheimer's disease. Alzheimers Dement. 7, 263–269. doi: 10.1016/j.jalz.2011.03.005, PMID: 21514250 PMC3312024

[ref41] Moretti R. Caruso P. Dal Ben M. Conti C. Gazzin S. Tiribelli C. (2017). Vitamin D, homocysteine, and folate in subcortical vascular dementia and Alzheimer dementia. Front. Aging Neurosci. 9:169. doi: 10.3389/fnagi.2017.00169, PMID: 28611659 PMC5447683

[ref42] Nägga K. Gustavsson A.-M. Stomrud E. Lindqvist D. van Westen D. Blennow K.. (2018). Increased midlife triglycerides predict brain β-amyloid and tau pathology 20 years later. Neurology 90, e73–e81. doi: 10.1212/WNL.0000000000004749, PMID: 29196581 PMC5754649

[ref43] NHLBI BioLINCC (2022). The BioLINCC handbook-biologic specimen and data repositories NIH NHLBI. Bethesda, Maryland, USA.

[ref44] Oda E. Oohara K. Abe A. Veeraveedu P. T. Watanabe K. Kato K.. (2006). The optimal cut-off point of C-reactive protein as an optional component of metabolic syndrome in Japan. Circ J 70, 384–388. doi: 10.1253/circj.70.384 16565552

[ref45] O'Leary D. H. Polak J. F. Wolfson S. K. Jr. Bond M. G. Bommer W. Sheth S.. (1991). Use of sonography to evaluate carotid atherosclerosis in the elderly. The cardiovascular health study. CHS collaborative research group. Stroke 22, 1155–1163. doi: 10.1161/01.STR.22.9.1155, PMID: 1926258

[ref46] Pietrzik C. U. Jaeger S. (2008). Functional role of lipoprotein receptors in Alzheimer's disease. Curr. Alzheimer Res. 5, 15–25. doi: 10.2174/156720508783884675 18288927

[ref47] Reitz C. Tang M.-X. Miller J. Green R. Luchsinger J. A. (2009). Plasma homocysteine and risk of mild cognitive impairment. Dement. Geriatr. Cogn. Disord. 27, 11–17. doi: 10.1159/000182421, PMID: 19088473 PMC2698462

[ref48] Rubio-Perez J. M. Morillas-Ruiz J. M. (2012). A review: inflammatory process in Alzheimer's disease, role of cytokines. Sci. World J. 2012, 1–15. doi: 10.1100/2012/756357, PMID: 22566778 PMC3330269

[ref49] SAS Institute Inc. (2014). Base SAS 9.4 procedures guide: statistical procedures SAS Institute. Cary, North Carolina, USA.

[ref50] Simon T. G. Trejo M. E. P. McClelland R. Bradley R. Blaha M. J. Zeb I.. (2018). Circulating Interleukin-6 is a biomarker for coronary atherosclerosis in nonalcoholic fatty liver disease: results from the multi-ethnic study of atherosclerosis. Int. J. Cardiol. 259, 198–204. doi: 10.1016/j.ijcard.2018.01.046, PMID: 29579601 PMC5875712

[ref51] Smith A. D. Refsum H. (2016). Homocysteine, B vitamins, and cognitive impairment. Annu. Rev. Nutr. 36, 211–239. doi: 10.1146/annurev-nutr-071715-050947, PMID: 27431367

[ref52] Taguchi A. (2009). Vascular factors in diabetes and Alzheimer's disease. J. Alzheimers Dis. 16, 859–864. doi: 10.3233/JAD-2009-0975 19387118

[ref53] Tahami Monfared A. A. Byrnes M. J. White L. A. Zhang Q. (2022). Alzheimer’s disease: epidemiology and clinical progression. Neurol. Ther. 11, 553–569. doi: 10.1007/s40120-022-00338-8, PMID: 35286590 PMC9095793

[ref54] Teng E. L. Hasegawa K. Homma A. Imai Y. Larson E. Graves A.. (1994). The cognitive abilities screening instrument (CASI): a practical test for cross-cultural epidemiological studies of dementia. Int. Psychogeriatr. 6, 45–58. doi: 10.1017/S1041610294001602, PMID: 8054493

[ref55] Waldstein S. R. Wendell C. R. (2010). Neurocognitive function and cardiovascular disease. J. Alzheimers Dis. 20, 833–842. doi: 10.3233/JAD-2010-091591 20413878

[ref56] Weiner M. W. Aisen P. S. Jack C. R. Jr. Jagust W. J. Trojanowski J. Q. Shaw L.. (2010). The Alzheimer's disease neuroimaging initiative: progress report and future plans. Alzheimers Dement. 6:e207. doi: 10.1016/j.jalz.2010.03.007 PMC2927112 20451868

[ref57] Woolf C. Slavin M. J. Draper B. Thomassen F. Kochan N. A. Reppermund S.. (2016). Can the clinical dementia rating scale identify mild cognitive impairment and predict cognitive and functional decline? Dement. Geriatr. Cogn. Disord. 41, 292–302. doi: 10.1159/000447057 27332560

[ref58] Yan R. T. Fernandes V. Yan A. T. Cushman M. Redheuil A. Tracy R.. (2010). Fibrinogen and left ventricular myocardial systolic function: the multi-ethnic study of atherosclerosis (MESA). Am. Heart J. 160, 479–486. doi: 10.1016/j.ahj.2010.06.001, PMID: 20826256 PMC2937158

[ref59] Yeboah J. Bertoni A. G. Herrington D. M. Post W. S. Burke G. L. (2011). Impaired fasting glucose and the risk of incident diabetes mellitus and cardiovascular events in an adult population: MESA (multi-ethnic study of atherosclerosis). J. Am. Coll. Cardiol. 58, 140–146. doi: 10.1016/j.jacc.2011.03.025, PMID: 21718910 PMC3146297

[ref60] Yoneyama K. Gjesdal O. Choi E.-Y. Wu C. O. Hundley W. G. Gomes A. S.. (2012). Age, sex, and hypertension-related remodeling influences left ventricular torsion assessed by tagged cardiac magnetic resonance in asymptomatic individuals: the multi-ethnic study of atherosclerosis. Circulation 126, 2481–2490. doi: 10.1161/CIRCULATIONAHA.112.093146, PMID: 23147172 PMC3558706

[ref61] Yu Z. Schneck M. Jacobs D. R. Jr. Liu K. Allison M. O'Leary D.. (2011). Association of carotid intima-media thickness with progression of urine albumin-creatinine ratios in the multi-ethnic study of atherosclerosis (MESA). Am. J. Kidney Dis. 57, 62–70. doi: 10.1053/j.ajkd.2010.08.014, PMID: 20974513

[ref62] Zlokovic B. V. Gottesman R. F. Bernstein K. E. Seshadri S. McKee A. Snyder H.. (2020). Vascular contributions to cognitive impairment and dementia (VCID): a report from the 2018 National Heart, Lung, and Blood Institute and National Institute of Neurological Disorders and Stroke workshop. Alzheimers Dement. 16, 1714–1733. doi: 10.1002/alz.12157, PMID: 33030307

