# Supplementary material for: Association of multiple metabolic and cardiovascular markers with the risk of cognitive decline and mortality in adults with Alzheimer’s disease and AD-related dementia or cognitive decline: a prospective cohort study
Source: Front Aging Neurosci. 2024 Apr 2;16:1361772. doi: 10.3389/fnagi.2024.1361772 (PMC11020085; doi:10.3389/fnagi.2024.1361772)
Supplement: Supplementary file 1 [file Table_1.pdf]

## Supplementary Table

**Supplementary** Table: Adjusted ORs of cognitive decline associated with MetSyn and covariates

| | Variables in model | OR | (95%CI) | |
|---|---|---|---|---|
| Mode1 | **MetSyn (yes vs. no)** | 1.32 | (1.14-1.53) | **<0.001** |
| | Age, (years) | 1.05 | (1.05-1.06) | **<0.001** |
| | Sex (Males vs. females) | 1.13 | (0.98-1.30) | 0.09 |
| Mode1b | **MetSyn (yes vs. no)** | 1.11 | (0.95-1.30) | 0.18 |
| | Age, (years) | 1.06 | (1.05-1.07) | **<0.001** |
| | Sex (Males vs. females) | 1.12 | (0.96-1.29) | 0.15 |
| | Race/ethnicity (Ref: White) | 1.83 | (1.72-1.95) | **<0.001** |
| Model1c | **MetSyn (yes vs. no)** | 1.02 | (0.86-1.19) | 0.86 |
| | Age, (years) | 1.06 | (1.05-1.07) | **<0.001** |
| | Sex (Males vs. females) | 1.00 | (0.86-1.17) | 0.97 |
| | Race/ethnicity (ref: White) | 1.62 | (1.52-1.73) | **<0.001** |
| | Education (Ref: <High School) | 0.30 | (0.26-0.35) | **<0.001** |
| Model 2 | **MetSyn (yes vs. no)** | 1.00 | (0.85-1.18) | 0.99 |
| | Age, (years) | 1.06 | (1.05-1.06) | **<0.001** |
| | Sex (Males vs. females) | 0.90 | (0.76-1.06) | 0.21 |
| | Race/ethnicity (ref: White) | 1.60 | (1.50-1.71) | **<0.001** |
| | Education (Ref: <High School) | 0.32 | (0.27-0.37) | **<0.001** |
| | Smoking (Ref: Never smoked) | 1.00 | (0.90-1.13) | 0.95 |
| | Physical activity (Ref: less than regular activity) | 1.03 | (0.86-1.23) | 0.74 |
| | Alcohol consumption (Ref: never use) | 0.73 | (0.66-0.81) | **<0.001** |
